# Association between triglyceride glucose and acute kidney injury in patients with acute myocardial infarction: a propensity score‑matched analysis

**DOI:** 10.1186/s12872-024-03864-5

**Published:** 2024-04-20

**Authors:** Dabei Cai, Tingting Xiao, Qianwen Chen, Qingqing Gu, Yu Wang, Yuan Ji, Ling Sun, Jun Wei, Qingjie Wang

**Affiliations:** 1 Department of Cardiology, the Affiliated Changzhou Second People’s Hospital of Nanjing Medical University, Changzhou, Jiangsu 213000 China; 2 Graduate School of Dalian Medical University, Dalian Medical University, Dalian, Liaoning 116000 China; 3 Department of Cardiovascular Surgery, the First Affiliated Hospital of Wannan Medical College, Wuhu, Anhui 241000 China; 4 Department of Cardiovascular Surgery, the Affiliated Hospital of Xuzhou Medical University, Xuzhou, 220005 China

**Keywords:** Triglyceride glucose index, Acute myocardial infarction, Acute kidney injury, Propensity score matching, Medical Information Mart for Intensive Care database

## Abstract

**Background:**

Acute kidney injury (AKI) in patients with acute myocardial infarction (AMI) often indicates a poor prognosis.

**Objective:**

This study aimed to investigate the association between the TyG index and the risk of AKI in patients with AMI.

**Methods:**

Data were taken from the Medical Information Mart for Intensive Care (MIMIC) database. A 1:3 propensity score (PS) was set to match patients in the AKI and non-AKI groups. Multivariate logistic regression analysis, restricted cubic spline (RCS) regression and subgroup analysis were performed to assess the association between TyG index and AKI.

**Results:**

Totally, 1831 AMI patients were included, of which 302 (15.6%) had AKI. The TyG level was higher in AKI patients than in non-AKI patients (9.30 ± 0.71 mg/mL vs. 9.03 ± 0.73 mg/mL, *P* < 0.001). Compared to the lowest quartile of TyG levels, quartiles 3 or 4 had a higher risk of AKI, respectively (Odds Ratio_model 4_ = 2.139, 95% Confidence Interval: 1.382–3.310, for quartile 4 vs. quartile 1, *P*_trend_ < 0.001). The risk of AKI increased by 34.4% when the TyG level increased by 1 S.D. (OR: 1.344, 95% CI: 1.150–1.570, *P* < 0.001). The TyG level was non-linearly associated with the risk of AKI in the population within a specified range. After 1:3 propensity score matching, the results were similar and the TyG level remained a risk factor for AKI in patients with AMI.

**Conclusion:**

High levels of TyG increase the risk of AKI in AMI patients. The TyG level is a predictor of AKI risk in AMI patients, and can be used for clinical management.

**Supplementary Information:**

The online version contains supplementary material available at 10.1186/s12872-024-03864-5.

## Introduction

As the most serious ischaemic heart disease, acute myocardial infarction (AMI) is recognized as a leading cause of cardiovascular disease (CVD) morbidity and mortality worldwide [1, 2]. AMI causes more than 24 million deaths in the United States and more than 4 million deaths in Europe and Northern Asia each year [3], accounting for more than one-third of all deaths in developed countries [4]. In recent decades, evidence-based therapies and lifestyle interventions have significantly reduced mortality from coronary heart disease [3]. However, CVD and AMI still brough a huge economic burden, heavier in low- and middle-income countries [5, 6]. In 2010 alone, the direct cost of hospitalization for myocardial infarction in the United States exceeded $450 billion [7].

Acute kidney injury (AKI) is a common and serious complication of AMI [8], usually caused by comorbid factors, hemodynamic instability, and the use of nephrotoxic medications. Studies have shown that the prevalence of AKI ranges from 7.1–29.3% [9–11]. AKI during hospitalization was independently associated with a higher in-hospital and long-term mortality after AMI [12–18].

As a newly recognized indicator of insulin resistance (IR), triglyceride-glucose (TyG) shows a large diagnostic and predictive value for diabetes than blood glucose [19]. IR patients are prone to a variety of metabolic disorders, such as hyperglycemia, dyslipidemia, and hypertension, all of which are strongly associated with adverse CVD outcomes [20]. For example, the TyG level has a stable prognostic value for CAD patients [21, 22]. It can be used to stratify risks and predict the prognosis in patients with acute coronary syndrome (ACS), and predict future major adverse cardiovascular events (MACE) in patients with diabetes combined with ACS independent of known cardiovascular risk factors [23]. Moreover, it is an independent risk factor for in-hospital mortality in patients with acute ST-elevation myocardial infarction, and a criterion for Mitral annular calcification [24]. Meanwhile, the TyG level is significantly associated with heart failure (HF) in AMI patients [25]. The TyG level is also positively correlated with the prognosis of patients with chronic HF and diabetes mellitus: a higher TyG index indicates a higher risk of cardiovascular death or rehospitalization due to HF [26]. In AMI patients, AKI may lead to a worse prognosis. Depsite the development of AKI risk prediction models in AMI patients [27–30], the relationship between the TyG level and the risk of AKI in patients with AMI is unclear.

Therefore, this study aimed to investigate the association between the TyG level and the AKI risk in AMI patients. Our findings may be depended on to design new strategies to manage AMI-related AKI.

## Materials and methods

### Data source

AMI patient data were obtained from the Medical Information Mart for Intensive Care (MIMIC) v1.4 and MIMIC-IV v2.2 databases. Use of the MIMIC database was approved by the Beth Israel Deaconess Medical Center and the MIT Institutional Review Board. Approval was obtained after application and completion of courses and testing (record IDs: 44,703,031 and 44,703,032). Informed consent was not required, because all patients’ information in the database was anonymized [31].

### Patient enrollment and data collection

Data extraction was programmed using Structured Query Language (SQL) in Navicat Premium (version 15.0.12). Patients with AMI were identified using ICD-9 and ICD-10 (International Classification of Diseases, Ninth and Tenth Revision) codes, and patients with AMI were identified using codes 41,000–41,092 and I21-I219. If the patient was admitted for multiple times, only the first admission was included. Exclusion criteria: (1) patients younger than 18 years or older than 90 years; (2) patients with incomplete test results of serum creatinine, glucose, and triglyceride; and (3) patients with data missed by more than 30%.

Clinical data were collected form eligible subjects, including demographics, comorbidities, vital signs, and laboratory parameters. Comorbidities included atrial fibrillation (AF), type 2 diabetes mellitus (T2DM), hypertension, chronic kidney disease, and obstructive sleep apnea (OSA). Vital signs were collected from the hospitalization records at the first admission, including heart rate (HR), respiratory rate (RR), temperature (T), systolic blood pressure (SBP), diastolic blood pressure (DBP), and mean blood pressure (MAP). Laboratory parameters were obtained from the first examination after hospitalization, including red blood cells (RBC), white blood cells (WBC), platelets, hemoglobin, hematocrit, hemoglobin, hematocrit, mean corpuscular volume (MCV), mean corpuscular hemoglobin volume (MCH), mean corpuscular hemoglobin concentration (MCHC), albumin, alanine aminotransferase (ALT), aspartate transaminase (AST), creatine kinase isoenzyme MB (CK-MB), troponin-T (TNT), total bilirubin (TB), alkaline phosphatase (AP), blood urea nitrogen (BUN), creatinine, fasting blood glucose (FBG), triglycerides (TG), high-density lipoprotein cholesterol (HDL-C), low-density lipoprotein cholesterol (LDL-C), lactate, total carbon dioxide (T-CO2), arterial carbon dioxide partial pressure (PaCO2), arterial oxygen saturation (SaO2), anion gap (AG), base excess (BE), bicarbonate, potassium, sodium, chloride, total calcium (T-calcium), phosphorus, magnesium, activated partial thromboplastin time (APTT), prothrombin time (PT), international normalized ratio (INR).

### Endpoint

The endpoint was AKI during hospitalization. The diagnosis of AKI was based on the latest international clinical practice guidelines for AKI [32], and accordance to any of the following three criteria: (a) creatinine rose ≥ 0.3 mg/dL (≥ 26.5 µmol/L) within 0 h; (b) creatinine rose to ≥ 1.5 times baseline within the 7 days; and (c) urine output < 0.5 mL/kg/hr over 6 h.

### Statistical analysis

Categorical variables were described by frequencies and percentages, and differences between groups were determined by the chi-squared test or Fisher’s exact test. Continuous variables were described by mean (± SD) or median and interquartile range (IQR), and differences between groups were determined by Student’s t-test or Mann-Whitney U test. Multivariate analyses (binary logistic regression) were performed to examine the association between the TyG level and the risk of AKI. Results were expressed as odds ratio (OR) and 95% confidence interval (95% CI). We validated the sample size to a rule that the number of outcome events should be ten per independent risk factor [33, 34]. In our study, the sample size was calculated to be 1000 patients or more to allow unbiased accommodation of less than ten predictors in a multivariable regression analysis under an assumed AKI incidence of at least 20%.

Propensity score matching (PSM) was used to minimize confounding bias and promote comparability between groups. We corrected for variables in Table 1 that differed in baseline characteristics and still had an effect on outcome events after multifactorial regression analysis. The final variables, including atrial fibrillation, creatinine, heart rate, and blood magnesium, were included in the model as matched variables. A 1:3 ratio, greedy nearest-neighbor matching, and no replacement were used. Matching was performed with a caliper of 0.2 on the PS to eliminate bias and compensate for the effect of potential confounders. Standardized mean difference (SMD) was used to compare baseline characteristics between the two groups.

**Table 1:** Baseline characteristics before and after matching

	Before Matching				Before Matching		
	Non-AKI (*n* = 1529)	AKI (*n* = 302)	*P*-value		Non-AKI (*n* = 838)	AKI (*n* = 301)	*P*-value
**Demographic**							
Age (Year)	64.08 (13.30)	65.61 (12.81)	0.066		64.70 (13.36)	65.64 (12.82)	0.293
Sex (Male)	1060 (69.3)	202 (66.9)	0.442		593 (70.8)	201 (66.8)	0.223
**Vital signs**							
HR (minˉ¹)	83.96 (17.25)	88.74 (19.72)	< 0.001		87.53 (18.46)	88.67 (19.72)	0.366
RR (minˉ¹)	18.27 (5.33)	19.04 (5.31)	0.023		18.81 (5.67)	19.03 (5.31)	0.552
SBP (mmHg)	124.68 (21.95)	120.96 (25.21)	0.009		123.54 (22.35)	121.10 (25.14)	0.117
DBP (mmHg)	71.23 (15.92)	70.04 (17.35)	0.242		70.92 (16.09)	70.12 (17.32)	0.473
MAP (mmHg)	89.06 (16.03)	87.01 (18.02)	0.047		88.47 (16.37)	87.11 (17.97)	0.228
T (°C)	36.60 [36.10, 36.90]	36.55 [36.10, 37.00]	0.635		36.60 [36.10, 36.90]	36.60 [36.10, 37.00]	0.433
**Medication (n%)**							
Aspirin	1333 (87.2)	263 (87.1)	1		721 (86.0)	262 (87.0)	0.736
Clopidogrel	942 (61.6)	162 (53.6)	0.012		512 (61.1)	162 (53.8)	0.033
RAASi	437 (28.6)	67 (22.2)	0.028		230 (27.4)	67 (22.3)	0.093
MRA	59 (3.9)	13 (4.3)	0.84		45 (5.4)	13 (4.3)	0.576
β-blocker	1266 (82.8)	238 (78.8)	0.116		681 (81.3)	238 (79.1)	0.458
**Comorbidities (n%)**							
Hypertension	546 (35.7)	101 (33.4)	0.492		293 (35.0)	101 (33.6)	0.711
T2DM	226 (14.8)	55 (18.2)	0.154		113 (13.5)	55 (18.3)	0.056
AF	215 (14.1)	62 (20.5)	0.005		163 (19.5)	62 (20.6)	0.731
CKD	121 (7.9)	34 (11.3)	0.073		77 (9.2)	34 (11.3)	0.345
OSA	51 (3.3)	14 (4.6)	0.344		23 (2.7)	14 (4.7)	0.158

Values are mean ± SD, n (%), or median (IOR).

HR, heart rata; RR, Respiratory rate; SBP, systolic blood pressure; DBP, diastolic blood pressure; MAP, mean arterial pressure; T, Temperature; RAASi, Renin-angiotensin-aldosterone System inhibitors; MRA, mineralocorticoid receptor antagonist AF, atrial fibrillation; T2DM, diabetes mellitus type 2; CKD, chronic kidney disease; OSA, Obstructive sleep apnea; AKI, acute kidney injury

The TyG index was calculated based on triglyceride (TG) and fasting blood glucose (FBG) concentrations using the formula: $\text{T}\text{y}\text{G}=\text{L}\text{n}\frac{\text{T}\text{G} (\text{m}\text{g}/\text{d}\text{L})\text{*} \text{F}\text{B}\text{G} (\text{m}\text{g}/\text{d}\text{L})}{2}$. We divided the population into four groups based on the magnitude of TyG levels. The first group was the reference group. To evaluate the relationship between TyG index and AKI risk, univariate and multivariate logistic regression analyses were performed before and after PSM. Model 1 included only TyG without any other adjustment. In model 2, sex, age and vital signs from Table 1 were added for adjustment. Model 3 was further adjusted for medications and comorbidities. Model 4 was additionally adjusted for laboratory test results. In addition, restricted cubic spline (RCS) regression was used to assess a possible nonlinear relationship between TyG level and AKI risk. Age, sex, atrial fibrillation, type 2 diabetes mellitus, and hypertension status were adjusted in the subgroup analysis.

R software (version 4.3.1) was used for statistical analysis, and GraphPad Prism (version 8.3.0) to generate graphs. All statistical tests were two-tailed, and *P* values less than 0.05 were considered statistically significant.

## Results

### Baseline characteristics

**Fig. 1:**
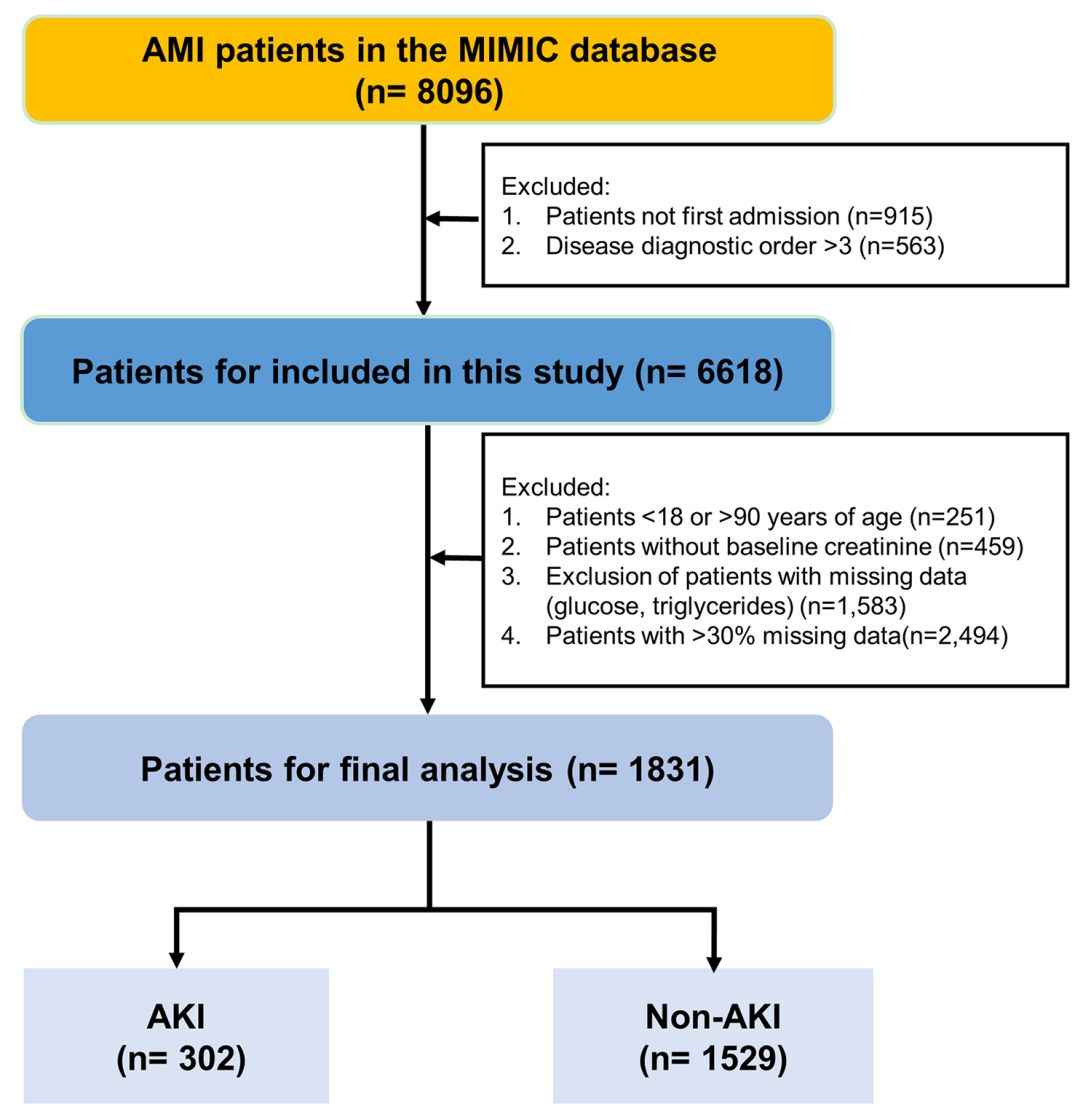
Flow diagram of the selection process of patients

A total of 1831 patients with AMI were included into this study (Fig. 1). AKI presented in 302 (15.6%) patients. Before PSM, age and sex ratios were not different between the AKI and non-AKI groups (*P* = 0.066, *P* = 0.442) (Table 1). According to the non-AKI group, the AKI group had a faster heart rate, and lower overall blood pressure, incidence of shortness of breath, consumption of clopidogrel and renin-angiotensin-aldosterone system (RAAS) inhibitors (*P*_all_ < 0.05), as well as a higher rate of AF (*P* = 0.005). Other baseline characteristics of the patients are shown in Tables 1 and 2.

**Table 2:** Laboratory test characteristics before and after matching

	Before Matching				Before Matching		
	Non-AKI (*n* = 1529)	AKI (*n* = 302)	*P*-value		Non-AKI (*n* = 838)	AKI (*n* = 301)	*P*-value
RBC (m/uL)	4.22 (0.70)	4.10 (0.77)	0.013		4.20 (0.74)	4.10 (0.77)	0.061
WBC (k/uL)	11.20 [8.70, 14.50]	12.35 [9.00, 16.40]	0.006		11.70 [8.80, 15.28]	12.30 [9.00, 16.40]	0.143
Platelet (100k/uL)	2.26 [1.87, 2.73.]	2.21 [1.77, 2.76]	0.166		2.27 [1.87, 2.77]	2.20 [1.77, 2.76]	0.076
Hemoglobin (g/dL)	12.74 (2.13)	12.35 (2.27)	0.005		12.66 (2.23)	12.36 (2.28)	0.043
Hematocrit (%)	37.66 (5.81)	36.75 (6.49)	0.015		37.43 (6.09)	36.75 (6.50)	0.101
MCV (fL)	89.45 (5.76)	90.03 (6.46)	0.115		89.39 (5.74)	90.01 (6.46)	0.118
MCH (pg)	30.28 (2.26)	30.15 (2.40)	0.366		30.22 (2.23)	30.16 (2.40)	0.688
MCHC (%)	33.85 (1.56)	33.52 (1.54)	0.001		33.80 (1.56)	33.53 (1.53)	0.009
Albumin (mg/dL)	3.27 (0.46)	3.15 (0.58)	< 0.001		3.26 (0.46)	3.15 (0.58)	0.002
ALT (IU/L)	57.00 [27.00, 88.00]	58.00 [27.00, 96.00]	0.145		57.00 [27.00, 88.00]	58.00 [27.00, 96.00]	0.26
AST (IU/L)	117.00 [46.00, 139.00]	117.00 [47.00, 208.50]	0.159		117.00 [45.00, 144.75]	117.00 [47.00, 198.00]	0.216
CKMB (IU/L)	63.00 [13.00, 138.00]	48.00 [10.00, 118.00]	0.172		59.00 [12.00, 119.00]	48.00 [10.00, 118.00]	0.514
TNT	2.81 [0.47, 4.00]	1.53 [0.27, 4.00]	0.044		2.49 [0.41, 4.00]	1.50 [0.27, 4.00]	0.125
TB (mg/dL)	0.80 [0.50, 1.70]	0.70 [0.40, 1.58]	0.045		0.70 [0.40, 1.70]	0.70 [0.40, 1.60]	0.147
AP (IU/L)	89.00 [65.00, 130.00]	84.00 [62.00, 128.00]	0.242		90.00 [65.00, 130.00]	84.00 [62.00, 128.00]	0.184
BUN (mg/dL)	18.00 [14.00, 25.00]	21.00 [15.00, 31.00]	< 0.001		19.00 [14.00, 27.00]	21.00 [15.00, 31.00]	0.001
Creatinine (mg/dL)	1.00 [0.80, 1.20]	1.20 [0.90, 1.67]	< 0.001		1.00 [0.80, 1.30]	1.20 [0.90, 1.60]	< 0.001
FBG (mg/dL)	137.00 [113.00, 181.00]	164.00 [123.00, 238.00]	< 0.001		139.00 [114.00, 189.75]	163.00 [123.00, 238.00]	< 0.001
TG (mg/dL)	110.00 [81.00, 162.00]	114.00 [84.00, 174.00]	0.1		106.00 [80.00, 156.75]	114.00 [84.00, 174.00]	0.015
HDL-C (mg/dL)	43.52 [37.00, 49.00]	43.52 [36.00, 44.75]	0.025		43.52 [37.00, 48.00]	43.52 [36.00, 45.00]	0.095
LDL-C (mg/dL)	96.80 [79.00, 111.00]	96.80 [73.50, 99.00]	0.068		96.80 [79.25, 110.00]	96.80 [75.00, 99.00]	0.13
Lactate (mg/dL)	2.50 [1.80, 2.50]	2.50 [1.50, 2.80]	0.084		2.50 [1.80, 2.50]	2.50 [1.50, 2.80]	0.156
T-CO_2_ (mEq/L)	25.00 [23.00, 25.00]	25.00 [21.00, 25.00]	< 0.001		25.00 [22.00, 25.00]	25.00 [21.00, 25.00]	0.005
SaO_2_ (%)	96.39 (5.08)	95.56 (6.25)	0.012		96.11 (5.37)	95.56 (6.26)	0.149
PCO2 (mmHg)	41.24 (6.86)	41.26 (8.17)	0.956		40.95 (7.01)	41.20 (8.11)	0.619
AG (mEq/L)	15.43 (3.60)	16.63 (4.36)	< 0.001		15.74 (3.82)	16.57 (4.27)	0.002
BE (mEq/L)	0.00 [-2.00, 1.00]	-1.00 [-6.00, 0.00]	< 0.001		0.00 [-3.00, 1.00]	-1.00 [-6.00, 0.00]	< 0.001
Bicarbonate (mg/dL)	23.10 (3.93)	21.68 (4.42)	< 0.001		22.87 (4.02)	21.69 (4.42)	< 0.001
Potassium (mEq/L)	4.18 (0.61)	4.26 (0.74)	0.036		4.21 (0.64)	4.26 (0.74)	0.293
Sodium (mEq/L)	138.04 (3.86)	137.69 (4.38)	0.164		138.17 (3.82)	137.70 (4.39)	0.078
Chloride (mEq/L)	103.11 (4.92)	102.72 (5.63)	0.228		103.19 (5.00)	102.77 (5.58)	0.225
T-Calcium (mEq/L)	8.59 (0.73)	8.36 (0.84)	< 0.001		8.56 (0.75)	8.37 (0.83)	< 0.001
Magnesium (mg/dL)	1.93 (0.31)	1.99 (0.37)	0.002		1.97 (0.33)	1.98 (0.36)	0.497
Phosphate (mg/dL)	3.40 [2.90, 4.00]	3.70 [3.00, 4.60]	< 0.001		3.60 [2.92, 4.10]	3.70 [3.00, 4.60]	0.007
INR	1.20 [1.10, 1.40]	1.20 [1.10, 1.37]	0.474		1.20 [1.10, 1.40]	1.20 [1.10, 1.30]	0.93
PT (s)	13.30 [12.30, 14.70]	13.35 [12.30, 14.90]	0.57		13.30 [12.40, 15.00]	13.30 [12.30, 14.90]	0.731
APTT (s)	36.00 [28.00, 62.00]	37.00 [28.00, 70.75]	0.547		36.00 [28.00, 61.75]	37.00 [28.00, 70.00]	0.491
TyG index [(mg/dL)^2^]	9.03 (0.73)	9.30 (0.71)	< 0.001		9.03 (0.70)	9.30 (0.71)	< 0.001

Values are mean ± SD, n (%), or median (IOR).

RBC, red blood cell; WBC, white blood cell; MCV, mean corpuscular volume; MCH, mean corpuscular hemoglobin; MCHC, mean corpuscular hemoglobin concentration; ALT, aspartate aminotransferase; AST, aspartate aminotransferase; CK, creatine kinase; CKMB, Creatine kinase isoenzyme MB; TNT, troponin-T; TB, Total Bilirubin; AP, Alkaline phosphatase; BUN, blood urea nitrogen; FBG, fasting blood glucose TG, triglyceride; HDL-C, High density lipoprotein cholesterol; LDL-C, Low density lipoprotein cholesterol; T-CO_2_, Total carbon dioxide; PaCO2, arterial partial pressure of carbon-dioxide; SaO_2_, AG, anion gap; BE, base excess; T-Calcium, Total Calcium; INR, International Normalized Ratio; PT, prothrombin time; APTT, activated partial prothrombin time;

### Relationship between TyG level and AKI risk

**Fig. 2:**
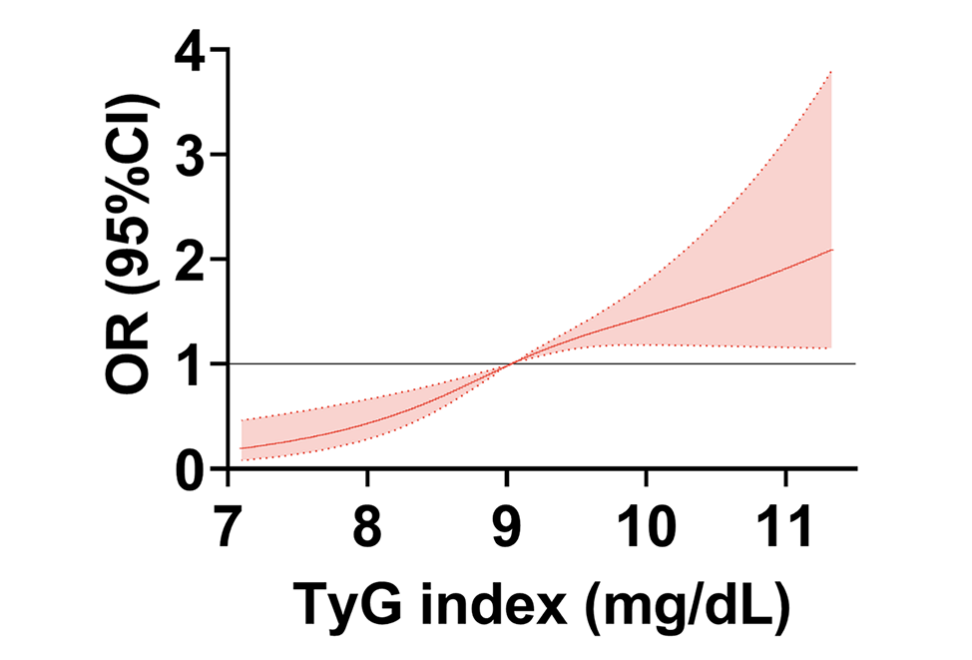
Single-volume restricted cubic spline regression showed a nonlinear relationship between TyG index and AKI. TyG: triglyceride glucose;

Before PSM, the mean TyG index was higher in the AKI group than in the non-AKI group (9.30 ± 0.71 mg/mL vs. 9.03 ± 0.73 mg/mL, *P* < 0.001). Table 3 shows the risk of AKI in patients with different quartiles of TyG levels. Four adjusted logistic models were constructed. Patients were divided into four categories according to TyG levels: Q1 (TyG ≤ 8.624 mg/mL), Q2 (8.624 mg/mL < TyG ≤ 9.030 mg/mL), Q3 (9.030 mg/mL < TyG ≤ 9.506 mg/mL), and Q4 (TyG > 9.506 mg/mL). A high TyG level increased the risk of AKI in adjusted terms (OR _adjusted_ = 1.499, 95% CI 1.211–1.856) (Table 3). Compared with that in Q1 with the lowest TyG levels, the risks of AKI increased significantly in Q3 and Q4 adjusted (OR _unadjusted_ = 2.535, 95% CI: 1.751–3.670, *P*_trend_ < 0.001 for Q 4 vs. Q 1; OR model_4_ = 2.139, 95% CI: 1.382–3.310, for Q4 vs. Q1, *P*_trend_ < 0.001) (Table 3). Specifically, the risk of AKI increased by 34.4% when the TyG level increased by 1 S.D. (OR = 1.344, 95%CI: 1.150–1.570, *P* < 0.001) (Table 3) after multivariate adjustment. A restricted RCS model revealed a non-linear relationship between TyG level and AKI risk. When the TyG level was greater than the cutoff value (approximately equal to 9 mg/mL), the AKI risk increased significantly with the TyG level (Fig. 2).

**Table 3:** TyG index levels and AKI risk in the entire population before propensity score matching

	Model1	Model2	Model3	Model4
TyG index	1.636 (1.382–1.936)	1.671 (1.401–1.992)	1.655 (1.38–1.986)	1.499 (1.211–1.856)
TyG index				
Q1	reference			
Q2	1.163 (0.771–1.754)	1.147 (0.758–1.737)	1.153 (0.759–1.752)	1.112 (0.722–1.712)
	0.472	0.515	0.503	0.629
Q3	2.200 (1.512–3.203)	2.249 (1.538–3.288)	2.291 (1.558–3.368)	2.004 (1.329–3.022)
	<0.001	<0.001	<0.001	0.001
Q4	2.535 (1.751–3.670)	2.578 (1.765–3.766)	2.526 (1.712–3.727)	2.139 (1.382–3.310)
	<0.001	<0.001	<0.001	0.001
*P* for trend	<0.001	<0.001	<0.001	<0.001
TyG index (*per 1 S.D.*)	1.432 (1.266–1.619)	1.454 (1.279–1.654)	1.444 (1.265–1.65)	1.344 (1.150–1.570)
	<0.001	<0.001	<0.001	<0.001

Model 1: Unadjusted

Model 2: Adjusted for sex, Age, HR, RR, SBP, DBP, MAP, T

Model 3: Model 2 + sex, Age, HR, RR, SBP, DBP, MAP, T, AF, CKD, T2DM, Hypertension, OSA, Aspirin, MRA, Beta, Clopidogrel, RAASi

Model 4: Model 3 + RBC, WBC, Platelet, Hemoglobin, Hematocrit, MCV, MCH, MCHC, Albumin, ALT, AST, AP, CKMB, TB, TNT, Creatinine, BUN, HDL-C, LDL-C, Bicarbonate, BE, Lactate, PCO_2_, T-CO_2_, SaO_2_, AG, Potassium, Sodium, Chloride, Phosphate, T-Calcium, Magnesium, PT, APTT, INR

### Subgroup analysis

**Fig. 3:**
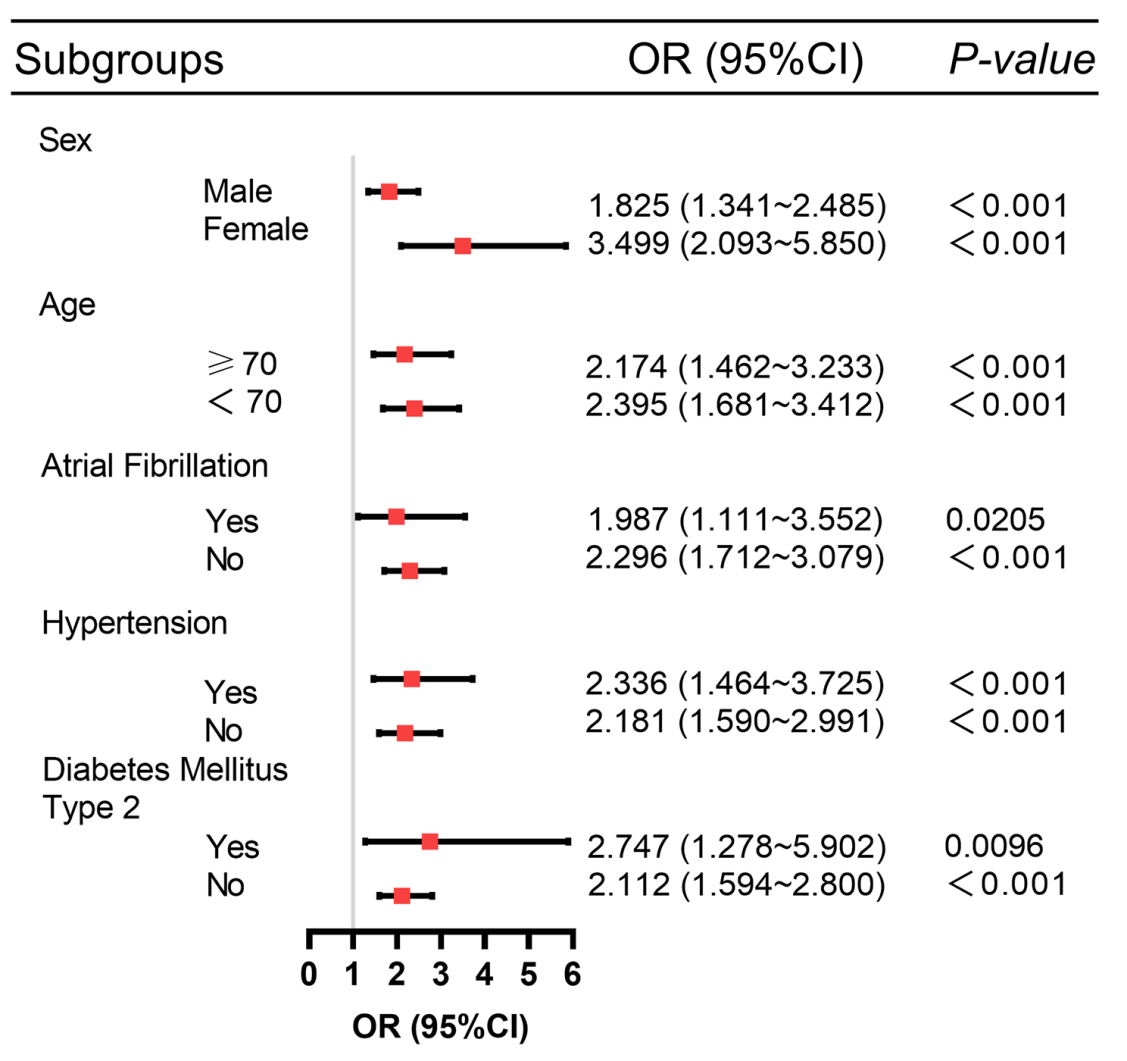
Propensity score matching before subgroup analysis of forest plots

A subgroup analysis was performed to confirm the relationship between TyG level and AKI risk in subgroups stratified by age, sex, atrial fibrillation, hypertension, and type 2 diabetes (Fig. 3). The study found that a higher TyG index was associated with an increased risk of AKI.

### PSM analysis

**Fig. 4:**
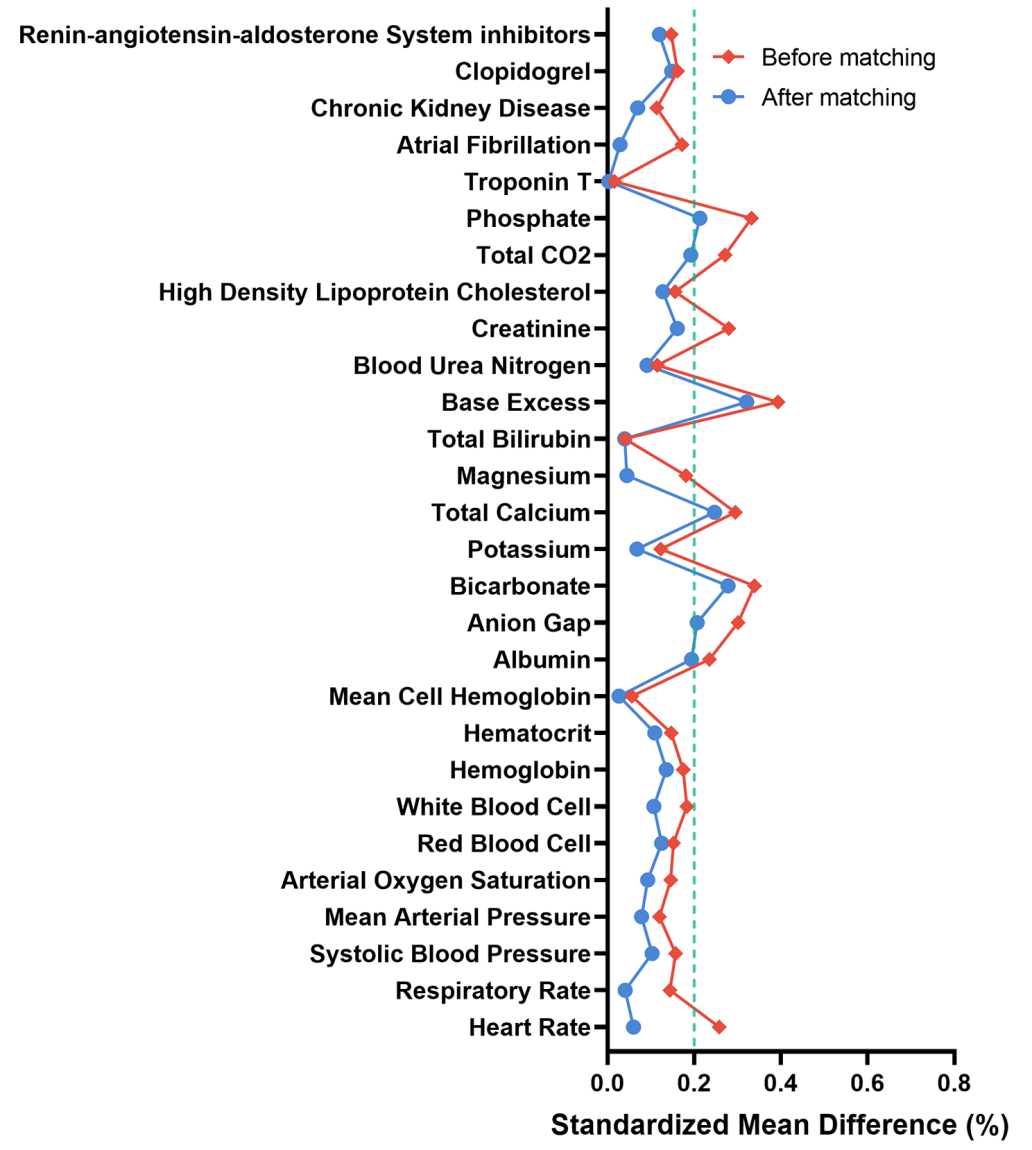
Balance checks of each variable after propensity score matching analysis. Standardized Mean differences of all the variables were illustrated

Finally, 838 patients without AKI were PS-matched to 301 patients with AKI. The balance between the groups was checked (Fig. 4). After matching, the variables were less significantly different from the baseline before matching. Tables 1 and 2 show the matched data characteristics between the AKI and non-AKI groups.

Paired groups underwent logistic regression analysis. After PS matching and multivariate adjustment, the risk of AKI increased by 36.3% when the TyG index increased by 1 S.D. (OR = 1.363, 95% CI: 1.153–1.611, *P* < 0.001) (Supplementary Table 1). TyG index similarly increased the risk of AKI in the adjusted cohort (OR _adjusted_ =1.546, 95% CI: 1.222–1.956); a higher TyG level was also associated with a higher risk of AKI in the adjusted matched cohort (OR _adjusted_ = 2.206, 95% CI: 1.388–3.504 for Q 4 vs. Q 1, *P*_trend_ = 0.001) (supplementary Table e1).

### Subgroup analysis

**Fig. 5:**
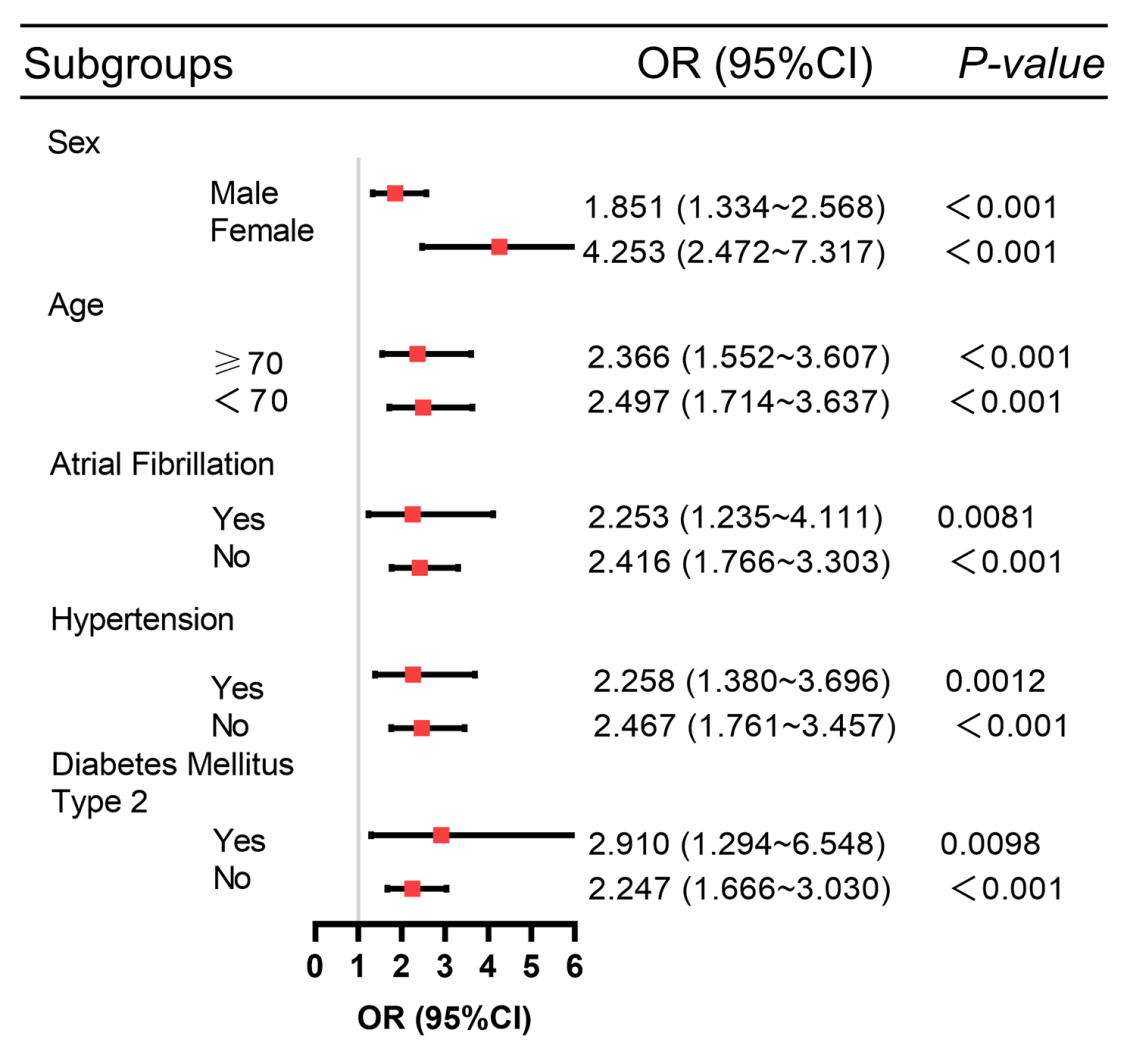
Propensity score matching after subgroup analysis of forest plots

Subgroup analyses were performed according to age, sex, atrial fibrillation, type 2 diabetes, and hypertension. It was further demonstrated that a high TyG index was associated with an increased risk of AKI in each subgroup (Fig. 5).

## Discussion

In the current study, we found that within a certain range, the risk of AKI increased with the TyG level in AMI patients. We found that the incidence of AKI in AMI patients was 15.6%, similar to those reported in previous studies [9–11]. Independent predictors of AKI included heart rate, base excess, total carbon dioxide, serum magnesium, atrial fibrillation and TyG index.

AMI patients are prone to concurrent AKI, and the specific pathological mechanism is not fully understood, but suspected to involve renal hypoperfusion, inflammation and endothelial injury [35, 36]. AKI also increases the incidence of renal and cardiovascular adverse events [37]. Atrial fibrillation has been reported to increase the risk of renal replacement therapy in patients with AMI, and subsequent in-hospital mortality [38]. Several studies have shown that lower or higher serum magnesium levels also increased the risk of AKI [39–43]. Indicators, such as heart rate, residual base and total carbon dioxide, may predict early shock, and are closely associated with the risks of renal and cardiovascular adverse events and death [44].

Through multivariate regression analysis and subgroup analysis, we found that the TyG index was independently associated with the risk of AKI, either before or after PSM. The TyG index can serve as a simple predictor for assessing the extent of IR, which is strongly associated with kidney damage [45]. The mechanism accounting for this relationship is not fully understood, but may be explained by the lipid accumulation in the kidney due to IR [46]. Renal lipid accumulation and subsequent lipotoxicity can damage renal structure and function [47–49], mainly manifested by an increase in oxidative stress mediated by hydrogen peroxide and superoxide [50–52]. In addition, nephrolipotoxicity not only causes AKI but also drives the progression of chronic kidney disease [53]. Some studies have reported that the visceral adiposity index [54] can also be considered as an indicator of IR, and that IR resistance is closely associated with various disorders of glucose and lipid metabolism, such as hyperglycaemia, dyslipidaemia and hypertension, mitral annular calcification and cardiovascular prognosis [55, 56]. Therefore, resolving IR or hyperinsulinemia is expected to effectively reduce the risk of AKI in AMI patients. However, there still lack studies at the cellular and animal levels and multicentre prospective clinical trials with large sample sizes and long-term follow-ups.

Our findings have profound implications in clinical practice. In our study, a higher TyG index was associated with an increased risk of AKI in a specific AMI population, suggesting that the TyG index may be a valuable tool for risk stratification and clinical management. To reduce the AKI risk associated with high TyG levels, a comprehensive risk management approach can be adopted, involving active management of cardiovascular risk factors, such as lipid control, body mass index, fasting glucose and glycated haemoglobin, and smoking. Regular monitoring and timely intervention in patients with elevated TyG index levels are essential to reduce the occurrence of adverse outcomes.

However, this study is limited in several aspects. First, the sample size of this study was small and all subjects were not followed up in a short or long term to further clarify the effects of TyG index on renal function. Therefore, future studies with larger sample sizes and longer follow-up periods should be performed to provide stronger evidence to support our findings. Second, the MIMIC database has a high quality, but lacks the data about some clinical characteristics, such as contrast imaging and contrast use in patients with AMI, so our study could not adjust for all potential confounders.

## Conclusions

A high TyG index level is associated with an increased risk of AKI in AMI patients. TyG index may be a valuable tool for risk classification and clinical management. Further studies are needed to confirm these results and determine the mechanism underlying the link between TyG index and AKI in AMI patients.

### Electronic supplementary material

Below is the link to the electronic supplementary material.

Supplementary Material 1

## Data Availability

The datasets presented in this study can be found in online repositories. The names of the repository/repositories and accession number(s) can be found below: https://physionet.org/content/mimiciii-demo/1.4/ and https://physionet.org/content/mimiciv/2.2/. In-hospital AKI diagnoses can also be accessed directly through the officially provided view codes https://github.com/MIT-LCP/mimic-code/.
