# Supplementary material for: Association between triglyceride glucose and acute kidney injury in patients with acute myocardial infarction: a propensity score‑matched analysis
Source: BMC Cardiovasc Disord. 2024 Apr 20;24:216. doi: 10.1186/s12872-024-03864-5 (PMC11031878; doi:10.1186/s12872-024-03864-5)
Supplement: Supplementary file 1 — Supplementary Material 1 [file 12872_2024_3864_MOESM1_ESM.docx]

Table 1. TyG index levels and AKI risk in the entire population after propensity score matching

|  | Model1 | Model2 | Model3 | Model4 |
| --- | --- | --- | --- | --- |
| TyG index | 1.704 (1.410-2.058) | 1.758 (1.445-2.139) | 1.737 (1.418-2.126) | 1.546 (1.222-1.956) |
| TyG |  |  |  |  |
| Q1 | reference |  |  |  |
| Q2 | 1.165 (0.765-1.775) | 1.165 (0.762-1.781) | 1.161 (0.756-1.783) | 1.157 (0.742-1.806) |
|  | 0.478 | 0.480 | 0.494 | 0.520 |
| Q3 | 2.243 (1.513-3.325) | 2.326 (1.562-3.463) | 2.412 (1.609-3.615) | 2.123 (1.372-3.283) |
|  | ＜0.001 | ＜0.001 | ＜0.001 | 0.001 |
| Q4 | 2.609 (1.767-3.852) | 2.694 (1.813-4.004) | 2.651 (1.762-3.987) | 2.206 (1.388-3.504) |
|  | ＜0.001 | ＜0.001 | ＜0.001 | 0.001 |
| *P* for trend | ＜0.001 | ＜0.001 | ＜0.001 | ＜0.001 |
| TyG (*per 1 s.d.*) | 1.461 (1.277-1.6709) | 1.494 (1.299-1.717) | 1.481 (1.282-1.71) | 1.363 (1.153-1.611) |
|  | ＜0.001 | ＜0.001 | ＜0.001 | ＜0.001 |

Model 1: Unadjusted

Model 2: Adjusted for sex, Age, HR, RR, SBP, DBP, MAP, T

Model 3: Model 2 + sex, Age, HR, RR, SBP, DBP, MAP, T, AF, CKD, T2DM, Hypertension, OSA, Aspirin, MRA, Beta, Clopidogrel, RAASi

Model 4: Model 3 + RBC, WBC, Platelet, Hemoglobin, Hematocrit, MCV, MCH, MCHC, Albumin, ALT, AST, AP, CKMB, TB, TNT, Creatinine, BUN, HDL-C, LDL-C, Bicarbonate, BE, Lactate, PCO_2_, T-CO_2_, SaO_2_, AG, Potassium, Sodium, Chloride, Phosphate, T-Calcium, Magnesium, PT, APTT, INR
